# A Preliminary Study of Effects of Channel Number and Location on the Repeatability of Motor Unit Number Index (MUNIX)

**DOI:** 10.3389/fneur.2020.00191

**Published:** 2020-03-17

**Authors:** Farong Gao, Yueying Cao, Chuan Zhang, Yingchun Zhang

**Affiliations:** ^1^School of Automation, Artificial Intelligence Institute, Hangzhou Dianzi University, Hangzhou, China; ^2^Department of Biomedical Engineering, University of Houston, Houston, TX, United States

**Keywords:** channel number, high-density, motor unit number index, repeatability, surface electromyography, innervation zone

## Abstract

Motor Unit Number Index (MUNIX) is a technique that provides a susceptive biomarker for monitoring innervation conditions in patients with neurodegenerative diseases. A satisfactory repeatability is essential for the interpretation of MUNIX results. This study aims to examine the effect of channel number and location on the repeatability of MUNIX. In this study, 128 channels of high-density surface electromyography (EMG) signals were recorded from the biceps brachii muscles of eight healthy participants, at 10, 20, 30, 40, 50, 60, 70, 80, and 100% of maximal voluntary contraction. The repeatability was defined by the coefficient of variation (CV) of MUNIX estimated from three experiment trials. Single-channel MUNIX (sMUNIX) was calculated on a channel-specific basis and a multi-channel MUNIX (mMUNIX) approach as the weighted average of multiple sMUNIX results. Results have shown (1) significantly improved repeatability with the proposed mMUNIX approach; (2) a higher variability of sMUNIX when the recording channel is positioned away from the innervation zone. Our results have demonstrated that (1) increasing the number of EMG channels and (2) placing recording channels close to the innervation zone (IZ) are effective methods to improve the repeatability of MUNIX. This study investigated two potential causes of MUNIX variations and provided novel perspectives to improve the repeatability, using high-density surface EMG. The mMUNIX technique proposed can serve as a promising tool for reliable neurodegeneration evaluation.

## Introduction

Motor Unit Number Index (MUNIX) has been accepted as a neurological tool for technically friendly indexing the number of functioning motor unit (MU) of target muscle (1). Being better tolerated, easier and quicker to perform than motor unit number estimation (MUNE), MUNIX has been proved an as reliable biomarker for assessing MU loss in different patient populations, including amyotrophic lateral sclerosis (ALS) (2), spinal cord injury (SCI) (3), multifocal motor neuropathy (MMN) (4), post-polio syndrome (5), stroke (6) and spinal muscular atrophy (SMA) (7). Specifically, studies have shown that MUNIX is capable of detecting motor neuron loss in early stages of ALS before the patient has obvious weakness (8).

A reproducible MUNIX is crucial to the acquisition of credible observations for interpretation. The repeatability of MUNIX can be affected by multiple factors, including the variation in electromyography (EMG) signals and electrode positioning. Variations in compound muscle action potential (CMAP) signals and surface EMG contraction signals, and randomness of surface interferential patterns (SIP) selection can affect MUNIX results. Furthermore, suboptimal electrode placement has been suggested as the most recurrent source of errors and systematic mistakes (9). The repeatability of MUNIX has been reported in both healthy and patient subjects, measured by coefficient of variation (CV), interclass correlation coefficients (ICC) and/or correlation coefficients (CC) (8–11). CV values up to 52.9% has been observed in healthy and ALS patients (10, 12–15). It is therefore necessary to find solutions to improve the repeatability of MUNIX; nonetheless limited effort has been made.

Recent advances of high-density surface EMG has enabled the non-invasive acquisition of abundant spatiotemporal information and consequently advanced analysis techniques (16–19). In this study, we aimed to employ high-density surface EMG measurements to examine the repeatability of MUNIX in relation to the number and location of recording channels. Specifically, a multi-channel MUNIX (mMUNIX) method was proposed to generate a more reproducible MU quantity index.

## Materials and Methods

### Participants and Consent

Eight healthy subjects (two females, mean age 27 ± 4 years) without history of neurological diseases were recruited at the University of Houston. Subjects were well-informed of the experiment procedure, potential risks of the study and gave written informed consent. The experiment protocol was approved by the University of Houston and University of Texas Health Science Center-Houston institutional review board.

### Experiment Protocol

**Figure 1:**
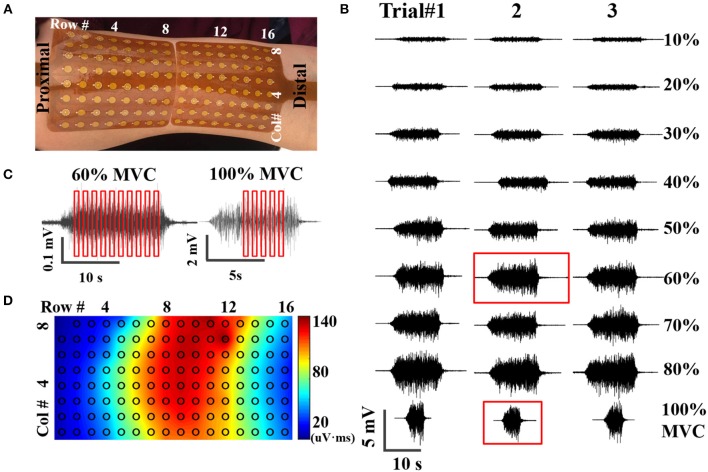
**(A)** placement of high-density surface EMG grids, **(B)** An example of EMG signals acquired at nine contraction levels from three trials, **(C)** Examples of SIP epoch selection at submaximal (60% MVC) and maximal (100% MVC) contractions, **(D)** Potential mapping from one representative CMAP recording.

The experiment procedure followed our previous study (20). Briefly, the biceps brachii muscle of the dominant arm was selected for MUNIX calculation. After skin preparation, two high-density surface EMG grids were placed adjacently to cover the muscle, as shown in Figure 1A. Each grid features an 8 by 8 surface electrode configuration, with an electrode diameter of 4.5 mm and an inter-electrode distance (IED) of 8.5 mm (TMSi, Enschede, the Netherlands). The reference electrode was placed on the medioepicondyle of the same arm and ground on the idle arm with a Velcro strap (TMSi, Enschede, the Netherlands). Subjects were seated in a mobile Biodex chair (Biodex, Shirley, NY) and instructed to perform three isometric elbow flexions at maximal voluntary contraction (MVC). Then three sets of experiment trials were performed. Each trial included contractions at 10, 20, 30, 40, 50, 60, 70, 80, and 100% MVC with visual feedback from a screen monitor and supramaximal compound muscle action potential (CMAP) elicited by electrical nerve stimulation. Rectangular stimulation with a pulse width of 0.2 ms was delivered to the proximal musculocutaneous nerve using a DS7 current stimulator (Digitimer Ltd, Welwyn Garden City, United Kingdom). The optimal stimulation site was determined by maximizing the CMAP response at a consistent stimulation intensity of 25 mA. Then stimulation intensity was increased in steps of 5 mA until no further increase in CMAP amplitude observed (21). Adequate interval was given between two consecutive contractions or stimulation to avoid muscle fatigue. All EMG signals were acquired via a 136 channel Refa amplifier (TMSi, Enschede, The Netherlands) at a sampling rate of 2,048 Hz, and stored for offline processing.

### MUNIX Calculation

Data analysis was performed using Matlab R2015 (The Mathworks, Natick, MA). Contraction EMG signals were bandpass filtered at 10–500 Hz and notch filtered at 60 Hz using second order Butterworth filters, as shown in Figure 1B. CMAP recording was high pass filtered at 1 Hz and notch filtered at 60 Hz, exampled by Figure 1D. Stimulation artifact was identified and suppressed as described in a previous study (22). Very briefly, the artifact was identified using a Savitzky-Golay filter and Otsu thresholding. Then the contaminated data points were replaced by a spline interpolation. As the compound action potential propagates, morphological and temporal alterations of the CMAP recordings were observed from different surface channels. Therefore, the high-density CMAP profile was obtained on a channel-specific basis by identifying the onset and offset of each CMAP measurement. SIP epochs were extracted from EMG trials at each contraction levels, with a length of 300 ms (614 data samples). Ten randomly selected epochs were extracted from each contraction level; whereas only 5 from 100% MVC because of the shorter duration of stable contractions at maximal force, as shown in Figure 1C. The final SIP pool consists of 85 different SIPs (8 submaximal contraction levels ^*^ 10 epochs/level + 1 contraction level of 100% MVC ^*^ 5 epochs/level). The SIP pool was employed to construct 10 different combinations of SIP epochs for MUNIX calculation by randomly selected one epoch in each level (SIP epochs at 100% MVC were used twice).

### Effect of Channel Number on MUNIX Repeatability

Single-channel MUNIX (sMUNIX) was calculated for each recording channel using the high-density SIP and CMAP profile. To evaluate the effect of channel number on the repeatability of MUNIX, a multi-channel MUNIX, denoted here as mMUNIX, was proposed. The method was inspired by a previous high-density MUNE approach (20, 23). Concretely, mMUNIX was calculated as the weighted average of multiple sMUNIX, with the weights defined as:

(1) $$\begin{array}{r} W\left(k\right)=\;\frac{A^{2}\left(k\right)}{\sum_{m=1}^{\text{N}}A^{2}\left(m\right)} \end{array}$$

where *A*(*k*) denotes the CMAP negative peak amplitude of the k-th channel, *W*(*k*) denotes its corresponding weight. mMUNIX was calculated, respectively, based on the *N* channels (*N* = 2, 4, 8, 16, 32, 64, and 128) with the top N largest CMAP amplitude.

### Effect of Channel Location on MUNIX Repeatability

**Figure 2:**
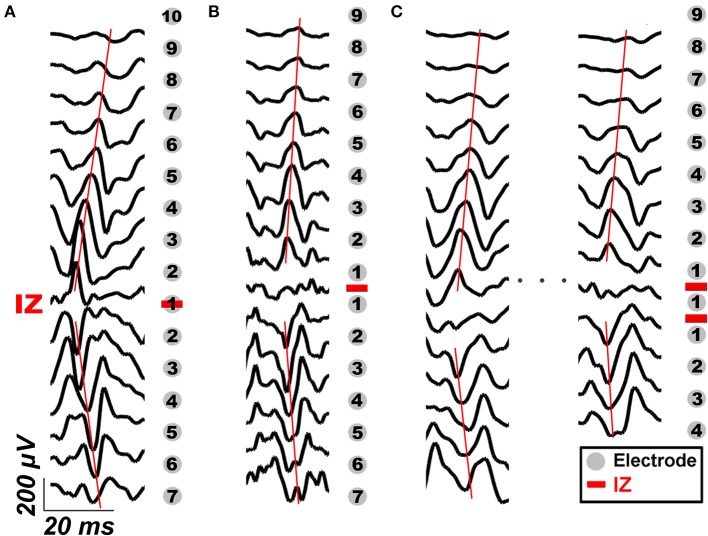
IZ identification and examples of channel labeling based on its distance to IZ in three representative cases: **(A)** one IZ located between two neighboring bipolar channels, **(B)** one IZ located on one bipolar channel, and **(C)** two distinct IZs. The gray dots mark the location of EMG electrodes, the red bars mark the location of IZ detected. The black traces are representative bipolar signals during voluntary contraction. The red lines mark the propagation of IZ.

As the CMAP area correlates with the MUNIX, the electrodes near IZ can often acquire larger CMAP response and consequently larger MUNIX (24). As innervation zone (IZ) closely related to the origin of EMG signals, the sMUNIX repeatability with respect to the IZ was also studied. The IZ was detected by treating each axial column as an evenly-spaced linear sensor array, and IZ was defined as the point of symmetry in the bipolar signals of each column, as shown in Figure 2 (25). If phase reversal was observed in two neighboring bipolar channels, the IZ was identified as the monopolar channel in the middle that contributes to both bipolar channels. If a bipolar channel with near-zero signal amplitude separated the signal phase reversal; the midpoint of the two monopolar channels which contribute to the attenuated bipolar channel was identified as the IZ (16, 26–28). Therefore, a spatial resolution of 4.25 mm (half of the inter-electrode spacing) and 8.5 mm was achieved for IZ detection in the axial and mediolateral directions, respectively. IZ mapping was determined at 20, 50, and 100% MVCs, which are the commonly force levels used for IZ detection (29). The channel label was defined by its minimal distance to the IZ on a column-basis, as shown in Figure 2. In Figure 2, the three labeled columns are to show three representative cases of IZ distributions: (1) one IZ located between two neighboring channels, (2) one IZ located on one channel, and (3) two distinct IZs. Concretely, a channel was labeled as *k* (*k* = 1, 2, …, 13) if the distance from the closest IZ was smaller than *k* IED but no less than *k* − 1 IED.

### Statistical Analysis

The consistency between mMUNIX and conventional MUNIX was evaluated by Pearson correlation coefficient (PCC). The variability of MUNIX was evaluated by the CV of all three experiment trials. To assess the effect of channel number on MUNIX repeatability, CV of sMUNIX (single-channel MUNIX) and mMUNIX (multi-channel MUNIX) were compared. To assess the repeatability of sMUNIX with respect to the IZ, the sMUNIX values was grouped and compared based on the channel label.

## Results

mMUNX and sMUNIX were successfully calculated for all eight subjects. The conventional MUNIX can be represented by the sMUNIX of the channel with largest CMAP response, which is also equivalent to the mMUNIX when *N* = 1. Table 1 summarizes the conventional MUNIX and mMUNIX results averaged across three trials and PCCs for all eight subjects. For conventional MUNIX, an average MUNIX of 103.8 ± 16.4 was obtained, ranging from 89.5 to 135.7. A very strong correlation (PCC > 0.98) between the conventional MUNIX and all mMUNIX results was observed. The mMUNIX value decreased when more channels were included for calculation.

**Table 1:** mMUNIX results (Mean and standard deviation) and PCCs of all subjects.

**Sub #**	***N* = 1**	**2**	**4**	**8**	**16**	**32**	**64**	**128**
1	89.5 (2.5)	88.3 (2.0)	87.4 (2.0)	86.9 (2.2)	85.9 (2.0)	84.4 (2.0)	80.1 (2.3)	73.1 (2.4)
2	92.0 (2.0)	91.9 (1.9)	92.0 (1.9)	91.0 (1.9)	88.9 (2.2)	84.8 (2.0)	77.5 (1.7)	70.9 (0.9)
3	135.7 (4.5)	136.2 (4.7)	135.7 (3.9)	134.7 (4.0)	133.5 (3.3)	128.8 (3.0)	119.5 (2.6)	107.4 (2.2)
4	85.3 (3.1)	85.5 (3.0)	85.3 (2.7)	84.3 (2.3)	83.2 (2.5)	80.0 (2.3)	73.7 (2.0)	66.8 (1.6)
5	108.0 (10.1)	107.8 (9.9)	107.2 (9.7)	106.4 (9.4)	105.1 (9.4)	102.1 (9.2)	95.5 (8.8)	86.3 (7.6)
6	104.1 (2.7)	103.9 (3.2)	103.4 (3.5)	101.3 (3.4)	97.4 (2.9)	92.9 (2.4)	87.7 (2.1)	80.8 (1.8)
7	99.8 (2.6)	98.8 (2.4)	98.6 (2.1)	98.0 (1.9)	96.1 (1.5)	92.3 (1.1)	86.3 (0.8)	78.4 (1.3)
8	115.7 (4.4)	115.1 (3.6)	113.7 (3.4)	110.8 (3.4)	107.9 (3.3)	104.4 (3.5)	99.6 (3.7)	91.8 (3.6)
Mean	103.8 (16.4)	103.4 (16.6)	102.9 (16.5)	101.7 (16.2)	99.8 (16.2)	96.2 (15.7)	90.0 (14.8)	81.9 (13.1)
PCC	–	0.9995	0.9985	0.9967	0.9918	0.9893	0.9922	0.9948

*Conventional MUNIX is reprensented by mMUNIX (N = 1)*.

The CVs of mMUNIX based on different channel number *N* were summarized in Table 2. Comparison between the repeatability of mMUNIX and conventional MUNIX was performed using a paired student's *t*-test, with *p*-values summarized. A significant lower CV of mMUNIX was observed. The overall CV decreased with the inclusion of more channels; yet in 3 of 8 subjects tested, the CV of mMUNIX increased when *N* was 64 or 128.

**Table 2:** CV of mMUNIX results with different channel number N.

**Sub #**	***N* = 1**	**2**	**4**	**8**	**16**	**32**	**64**	**128**
1	3.79	3.12	2.99	3.03	3.08	3.37	3.69	3.88
2	3.62	3.45	3.16	2.76	3.01	2.88	2.69	2.35
3	2.84	2.32	2.31	2.52	2.37	2.42	2.82	3.22
4	2.41	2.22	2.14	2.12	2.52	2.37	2.23	1.20
5	9.39	9.21	9.01	8.85	8.92	8.99	9.26	8.83
6	2.60	2.44	2.13	1.89	1.54	1.21	0.97	1.68
7	4.75	4.24	3.74	3.95	3.82	3.61	3.44	3.11
8	3.12	3.18	2.98	2.92	2.54	1.87	1.46	1.30
Mean	4.06	3.77	3.56	3.50	3.48	3.33	3.32	3.20
*p*	N/A	0.0052	0.0004	0.0001	0.0007	0.0013	0.0106	0.0078

**Figure 3:**
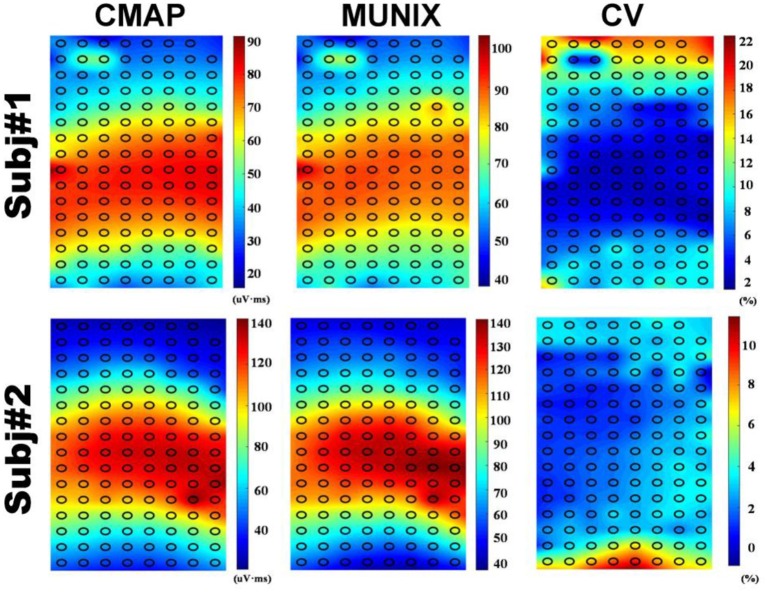
CMAP **(Left)**, MUNIX **(Middle)**, and CV **(Right)** mappings from two representative subjects.

Figure 3 shows the sMUNIX, CMAP area and CV mapping in two representative subjects. The sMUNIX mapping tends to correlate well with the CMAP area. CV mapping suggested relatively stable sMUNIX near the IZ regions yet more variable observations away from the IZ. Table 3 summarizes the results of CV of sMUNIX with respect to its distance to the IZ. Analysis showed higher sMUNIX CV in channels further from the IZ, yet no statistical signficant difference was observed after Benjamini-Hochberg correction. A larger CV variation was observed in sMUNIX of channels far away from the IZ.

**Table 3:** The CVs of mMUNIX results based on its distance from IZ.

**MVC level**	**20%**		**50%**		**100%**	
	**Mean**	**Std**	**Mean**	**Std**	**Mean**	**Std**
k = 1	3.53	2.49	3.56	2.46	3.57	2.49
2	3.59	2.74	3.57	2.78	3.6	2.69
3	4.13	3.01	4.06	3.15	4.06	3.1
4	4.7	3.14	4.57	3.31	4.65	3.3
5	5.94	3.61	5.89	3.82	5.69	3.77
6	7.1	4.31	7.57	4.85	7.35	4.57
7	8.43	5.62	8.62	5.13	8.37	5.18
8	10.26	7.37	10.37	7.04	10.44	7.08
9	9.79	8.45	9.25	7.35	9.17	7.49
10	7.78	7.52	7.75	6.82	7.86	6.76
11	9.59	8.4	11.21	8.06	10.4	8.7
12	11.32	8.04	12.96	10.19	13.54	9.37

*k is the distance label*.

## Discussion

In this study, MUNIX was evaluated in eight healthy subjects using high-density surface EMG, and results were consistent with previous studies (1, 8, 15, 30). A novel mMUNIX method has been proposed, by including additional recording channels and taking into account spatiotemporal EMG information. Our results suggested a high consistency between mMUNIX and conventional MUNIX, whereas mMUNIX was significantly more repeatable. Furthermore, sMUNIX estimations showed a relatively stable MUNIX estimation in a wide range of recording site, albeit the repeatability decreased when moving the channel position away from IZ.

Limited effort has been made to improve the repeatability of MUNIX. Ahn et al. employed a digital instrument to improve the MUNIX reproducibility by reducing the variations in SIP signals (31). Peng et al. demonstrated that the inclusion of additional SIP epochs at lower contraction levels can significantly improve the repeatability (20). Bezerra et al. found that the averaging across multiple measurements could generate more repeatable MUNIX (32). To control for the experience-related variations and improve the automation of MUNIX calculation, in this study, the MUNIX repeatability was evaluated by taking into consideration all three previously proposed methods. This explains that the relatively low trial-to-trial CV compared to previously reported results (9, 33).

In this study, the performance of mMUNIX have been assessed by PCC and CV. The strong correlation (all >0.98) between mMUNIX and standard MUNIX, in addition to the significantly reduced variability, has evidenced the validity of proposed mMUNIX technique. The improved repeatability of mMUNIX may be attributed to the addition of more spatiotemporal EMG information from a broader muscle area, whereas conventional MUNIX is performed with only one EMG channel positioned at where the largest CMAP is obtained. Additive myoelectric information is expected to provide a more comprehensive sampling of the motor unit information and therefore benefit MUNIX repeatability. The mMUNIX can be clinically performed by searching for the *N* locations with the top *N* largest CMAP response, and calculating the weighted average of the *N* sMUNIX values. As MUNIX stands out due to its simplicity in implementation, using more channels can complicate the experiment protocol and system demands. This remains a trade-off between improving the repeatability and the inclusion of more channels. It is also interesting to note that in 3 of 8 subjects tested, the CV of mMUNIX increased when *N* was 64 or 128, i.e., a large number of EMG channels were included. This could be explained in part by the noise introduced by channels that were positioned outside the target muscle region.

Previous high-density surface EMG based MUNE methods defined the weight by the size of the single motor unit potential (SMUP) rather than the CMAP (20), as MUNE is often sensitive to the SMUP estimation (34). However, in MUNIX calculation, no direct estimation of SMUP size was provided. Therefore, assigning weight based on motor unit size is not feasible. We have found that weights determined by the CMAP and root-mean-square of SIPs were similar while CMAP often provide more stable weight estimations (Data not shown).

The repeatability of MUNIX at non-optimal locations was also evaluated. Conventional MUNIX requires the placement of recording electrode at the surface location where the maximal CMAP was observed, which correlates with the region where neuromuscular junctions, indicated by IZ, are densely distributed. As the CMAP area correlated with the MUNIX, the electrodes near IZ can often acquire larger CMAP response and consequently larger MUNIX. We have observed an increased variability of sMUNIX when moving further away from the IZ region, which may also explain the increased variations of mMUNIX when N is very large. However, the repeatability of sMUNIX is not very different unless the electrode is positioned very far from the IZ. As the sample size is relatively small, it is possible to achieve the level of significance by increasing the subject size. Our results suggest that rather than searching for the optimal site with maximal CMAP, suboptimal placement close to the IZ may also provide reasonable MUNIX estimation with similar level of repeatability, as shown in Figure 3. The consistency of electrode positioning can be ensured by anatomical landmarks. The similar mappings of CMAP amplitude and MUNIX estimation of these subjects suggested that MUNIX estimation relies largely on the size of the CMAP, which is consistent with previous findings (8). The mappings of CMAP amplitude and MUNIX CV have shown a very interesting negative correlation, which corresponds with our results that MUNIX variability increases with the electrode-IZ distance. However, it should be noted the study was only tested in healthy participants, whether similar observation holds under pathophysiological conditions requires further study. Moreover, the three trials in this study were performed without removal and re-attaching the electrode grids, which also in part explained the small trial-to-trial variability. The variations of mMUNIX across different visits require further studies.

A correlation between MUNIX value and with the size of CMAP area was observed (8, 35, 36). However, MUNIX can provide more information and is proved more sensitive than CMAP alone (1, 4, 36). It had to be underlined that MUNIX does not estimate the actual quantity of existent MUs, but more of an “index” that related to the number of motor neurons (30, 36). Although not carrying physiologically meaning, MUNIX provides a reliable biomarker to detect neurodegenerative diseases.

## Data Availability Statement

The datasets generated for this study are available on request to the corresponding author.

## Ethics Statement

The studies involving human participants were reviewed and approved by University of Houston IRB. The patients/participants provided their written informed consent to participate in this study.

## Author Contributions

FG, YC, CZ, and YZ: experiment design and discussion and the final approval of the manuscript. YC and CZ: data collection. FG, YC, and CZ: data analysis. YC, CZ, and YZ: manuscript draft.

### Conflict of Interest

The authors declare that the research was conducted in the absence of any commercial or financial relationships that could be construed as a potential conflict of interest.
